# 1*H*-1,2,4-Triazol-4-ium 4-nitro­benzene­sulfonate monohydrate

**DOI:** 10.1107/S1600536811036774

**Published:** 2011-09-17

**Authors:** Madhukar Hemamalini, Ibrahim Abdul Razak, Hoong-Kun Fun

**Affiliations:** a X-ray Crystallography Unit, School of Physics, Universiti Sains Malaysia, 11800 USM, Penang, Malaysia

## Abstract

In the 4-nitro­benzene sulfonate anion of the title compound, C_2_H_4_N_3_^+^·C_6_H_4_NO_5_S^−^·H_2_O, the nitro group is slightly twisted from the plane of the benzene ring [dihedral angle = 2.8 (3)°]. In the crystal, the three components are linked *via* N—H⋯O, O—H⋯N, O—H⋯O and C—H⋯O hydrogen bonds, forming a two-dimensional network parallel to the *bc* plane. A short inter­molecular O⋯N contact of 2.872 (3) Å is also observed between the nitro and sulfonate groups.

## Related literature

For details and applications of aromatic sulfonates, see: Yachi *et al.* (1989 ▶); Spungin *et al.* (1992 ▶); Jiang *et al.* (1990 ▶); Narayanan & Krakow (1983 ▶).
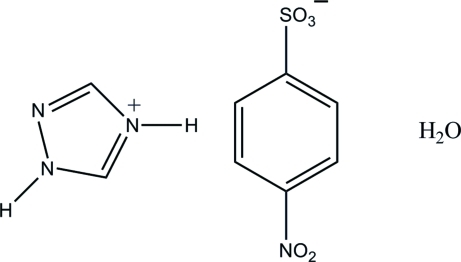


## Experimental

### 

#### Crystal data

C_2_H_4_N_3_^+^·C_6_H_4_NO_5_S^−^·H_2_O
*M* *_r_* = 290.26
Monoclinic, 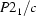

*a* = 14.0931 (13) Å
*b* = 6.4859 (6) Å
*c* = 14.5707 (14) Å
β = 117.182 (2)°
*V* = 1184.77 (19) Å^3^
*Z* = 4
Mo *K*α radiation
μ = 0.31 mm^−1^
*T* = 296 K
0.41 × 0.28 × 0.05 mm

#### Data collection

Bruker APEXII DUO CCD area-detector diffractometer
Absorption correction: multi-scan (*SADABS*; Bruker, 2009 ▶) *T*_min_ = 0.885, *T*_max_ = 0.986
10925 measured reflections
2692 independent reflections
2136 reflections with *I* > 2σ(*I*)
*R*_int_ = 0.038

#### Refinement

*R*[*F* ^2^ > 2σ(*F* ^2^)] = 0.043
*wR*(*F* ^2^) = 0.144
*S* = 1.07
2692 reflections
188 parameters
3 restraints
H atoms treated by a mixture of independent and constrained refinement
Δρ_max_ = 0.27 e Å^−3^
Δρ_min_ = −0.37 e Å^−3^

### 

Data collection: *APEX2* (Bruker, 2009 ▶); cell refinement: *SAINT* (Bruker, 2009 ▶); data reduction: *SAINT*; program(s) used to solve structure: *SHELXTL* (Sheldrick, 2008 ▶); program(s) used to refine structure: *SHELXTL*; molecular graphics: *SHELXTL*; software used to prepare material for publication: *SHELXTL* and *PLATON* (Spek, 2009 ▶).

## Supplementary Material

Crystal structure: contains datablock(s) global, I. DOI: 10.1107/S1600536811036774/is2774sup1.cif

Structure factors: contains datablock(s) I. DOI: 10.1107/S1600536811036774/is2774Isup2.hkl

Supplementary material file. DOI: 10.1107/S1600536811036774/is2774Isup3.cml

Additional supplementary materials:  crystallographic information; 3D view; checkCIF report

## Figures and Tables

**Table 1:** Hydrogen-bond geometry (Å, °)

*D*—H⋯*A*	*D*—H	H⋯*A*	*D*⋯*A*	*D*—H⋯*A*
N4—H1*NA*⋯O5^i^	0.88 (4)	1.88 (4)	2.744 (3)	169 (2)
O1*W*—H1*W*⋯N3	0.91 (4)	2.17 (4)	3.041 (3)	160 (4)
N2—H1*NB*⋯O1*W*^ii^	0.86 (4)	1.84 (4)	2.692 (3)	171 (3)
O1*W*—H2*W*⋯O3^iii^	0.95 (4)	1.86 (4)	2.774 (3)	161 (5)
C7—H7*A*⋯O4^iv^	0.93	2.36	3.063 (3)	132
C8—H8*A*⋯O1	0.93	2.54	3.186 (4)	126

Symmetry codes: (i) 
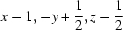
; (ii) 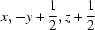; (iii) 
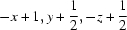
; (iv) 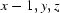.

**Fig. 1.**
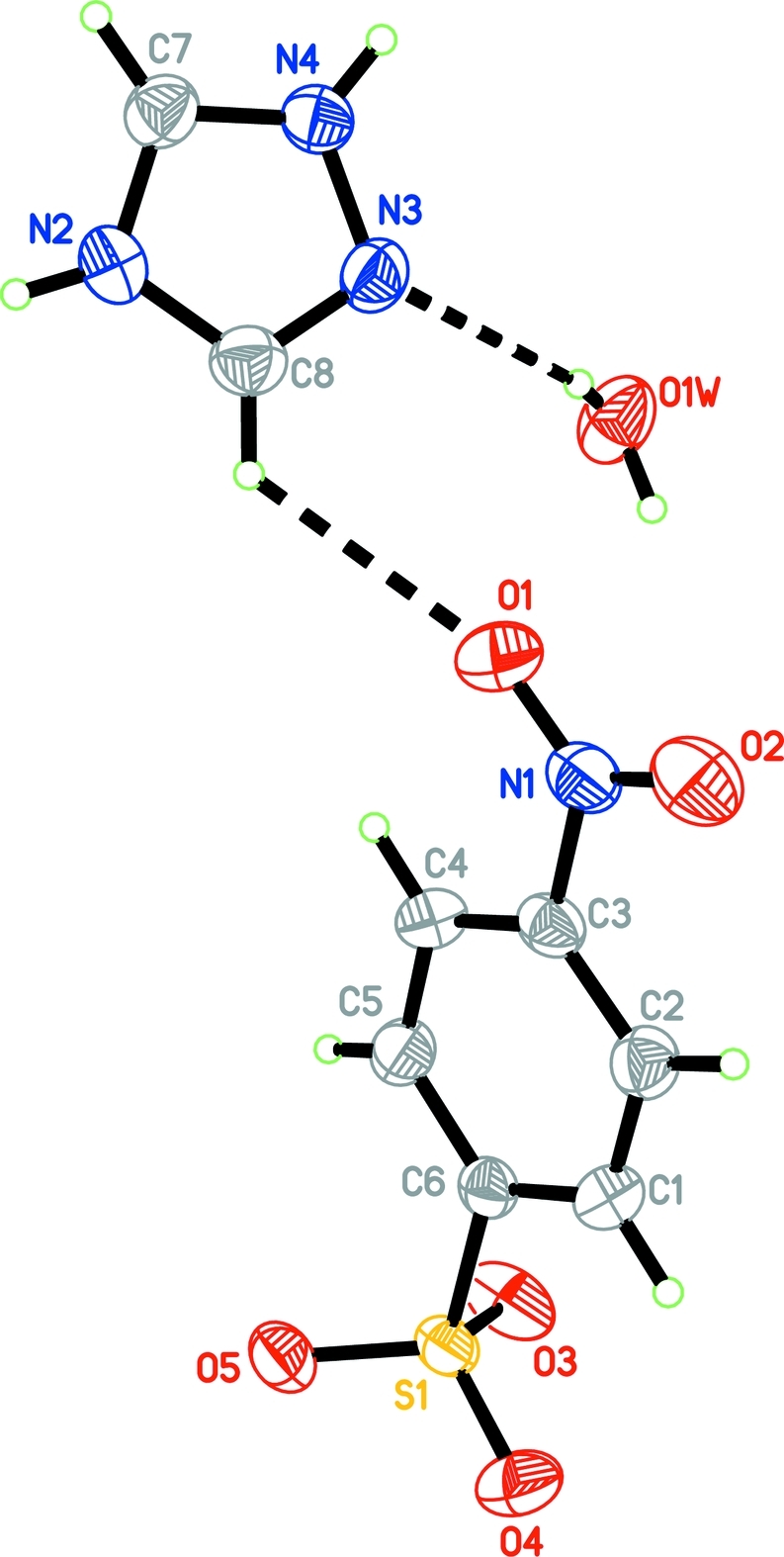
The asymmetric unit of the title compound, showing 50% probability displacement ellipsoids. Intermolecular O—H···N and C—H···O hydrogen bonds are shown by dashed lines.

**Fig. 2.**
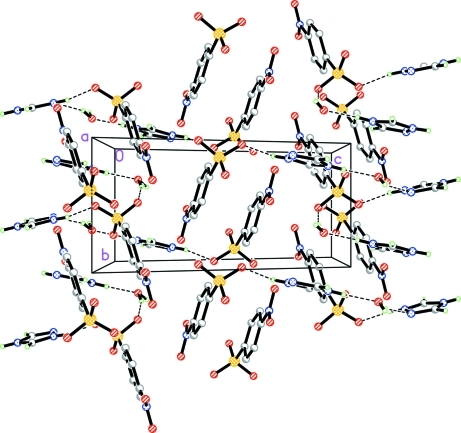
The crystal packing of the title compound. Dashed lines represent hydrogen bonds.

## supplementary crystallographic information

### Comment

In recent years, there has been of great interest in the design and utilization of 1,2,4-triazole and its derivatives in coordination and biological chemistry for they represent the simple small molecular ligands.  Aromatic sulfonates are used in monitoring the merging of lipids (Yachi *et al.*, 1989) and in many other fields (Spungin *et al.*, 1992; Jiang *et al.*, 1990; Narayanan & Krakow, 1983). An X-ray study of the title compound was undertaken in order to determine its crystal and molecular structure owing to the biological importance of its analogues. The molecular structure of the title compound (I).

The asymmetric unit of the title compound, (Fig. 1), contains a protonated 1,2,4-triazolinium cation, a 4-nitrobenzenesulfonate anion and a water molecule. In the  4-nitrobenzenesulfonate anion, the nitro and sulfonate groups are twisted slightly from the ring to which they are attached with the dihedral angles between the O1/O2/N1 and C1–C6 planes, and the S1/O3/O5 and C1–C6 planes being 2.8 (3) and 88.85 (13)°, respectively.

In the crystal structure, (Fig. 2), the ion pairs and water molecules are linked *via* intermolecular N—H···O, O—H···N, O—H···O and C—H···O hydrogen bonds (Table 1), forming two-dimensional networks parallel to (100). A short O···N contact of 2.87 Å is also observed.

### Experimental

A methanol solution (20 ml) of 1-(*p*-Nitrobenzenesulfonayl)-1*H*-1,2,4-triazole (63.55 mg, Aldrich) was warmed over a heating magnetic stirrer for 15 minutes. The resulting solution was allowed to cool slowly at room temperature. Crystals of the title compound appeared from the mother liquor after a few days.

### Refinement

Atoms H1NA and H1NB were located in a difference Fourier map and refined freely [N—H = 0.86 (3)–0.87 (3) Å]. Atoms H1W and H2W were also located in a difference map and were refined with restraints of bond lengths and angles [O—H = 0.917 (18)–0.950 (18) Å and H2W—O1W—H1W = 110 (3)°]. The remaining H atoms were positioned geometrically (C—H = 0.93 Å) and were refined using a riding model, with *U*_iso_(H) = 1.2 *U*_eq_(C).

### Crystal data

**Table:**

C_2_H_4_N_3_^+^·C_6_H_4_NO_5_S^−^·H_2_O	*F*(000) = 600
*M**_r_* = 290.26	*D*_x_ = 1.627 Mg m^−^^3^
Monoclinic, *P*2_1_/*c*	Mo *K*α radiation, λ = 0.71073 Å
Hall symbol: -P 2ybc	Cell parameters from 3747 reflections
*a* = 14.0931 (13) Å	θ = 2.8–30.9°
*b* = 6.4859 (6) Å	µ = 0.31 mm^−^^1^
*c* = 14.5707 (14) Å	*T* = 296 K
β = 117.182 (2)°	Block, colourless
*V* = 1184.77 (19) Å^3^	0.41 × 0.28 × 0.05 mm
*Z* = 4	

### Data collection

**Table:**

Bruker APEXII DUO CCD area-detector diffractometer	2692 independent reflections
Radiation source: fine-focus sealed tube	2136 reflections with *I* > 2σ(*I*)
graphite	*R*_int_ = 0.038
φ and ω scans	θ_max_ = 27.5°, θ_min_ = 1.6°
Absorption correction: multi-scan (*SADABS*; Bruker, 2009)	*h* = −18→16
*T*_min_ = 0.885, *T*_max_ = 0.986	*k* = −8→8
10925 measured reflections	*l* = −18→18

### Refinement

**Table:**

Refinement on *F*^2^	Primary atom site location: structure-invariant direct methods
Least-squares matrix: full	Secondary atom site location: difference Fourier map
*R*[*F*^2^ > 2σ(*F*^2^)] = 0.043	Hydrogen site location: inferred from neighbouring sites
*wR*(*F*^2^) = 0.144	H atoms treated by a mixture of independent and constrained refinement
*S* = 1.07	*w* = 1/[σ^2^(*F*_o_^2^) + (0.0862*P*)^2^ + 0.2559*P*] where *P* = (*F*_o_^2^ + 2*F*_c_^2^)/3
2692 reflections	(Δ/σ)_max_ = 0.001
188 parameters	Δρ_max_ = 0.27 e Å^−^^3^
3 restraints	Δρ_min_ = −0.37 e Å^−^^3^

### Special details

**Table:**

Geometry. All s.u.'s (except the s.u. in the dihedral angle between two l.s. planes) are estimated using the full covariance matrix. The cell s.u.'s are taken into account individually in the estimation of s.u.'s in distances, angles and torsion angles; correlations between s.u.'s in cell parameters are only used when they are defined by crystal symmetry. An approximate (isotropic) treatment of cell s.u.'s is used for estimating s.u.'s involving l.s. planes.
Refinement. Refinement of F^2^ against ALL reflections. The weighted R-factor wR and goodness of fit S are based on F^2^, conventional R-factors R are based on F, with F set to zero for negative F^2^. The threshold expression of F^2^ > 2σ(F^2^) is used only for calculating R-factors(gt) etc. and is not relevant to the choice of reflections for refinement. R-factors based on F^2^ are statistically about twice as large as those based on F, and R- factors based on ALL data will be even larger.

### Fractional atomic coordinates and isotropic or equivalent isotropic displacement parameters (Å^2^)

**Table:**

	*x*	*y*	*z*	*U*_iso_*/*U*_eq_	
O1W	0.09777 (16)	0.2748 (3)	0.10622 (14)	0.0521 (5)	
H2W	0.165 (2)	0.336 (7)	0.125 (4)	0.126 (16)*	
H1W	0.066 (3)	0.338 (7)	0.141 (3)	0.130 (18)*	
O3	0.72455 (14)	−0.0217 (3)	0.38351 (14)	0.0535 (5)	
O4	0.83055 (14)	0.2569 (3)	0.49177 (16)	0.0520 (5)	
O5	0.75198 (13)	0.0065 (3)	0.55875 (13)	0.0441 (4)	
N1	0.34545 (15)	0.6163 (3)	0.32475 (15)	0.0384 (5)	
C1	0.62491 (17)	0.4703 (4)	0.39475 (18)	0.0357 (5)	
H1A	0.6865	0.5296	0.3982	0.043*	
C2	0.53180 (18)	0.5842 (3)	0.35933 (18)	0.0360 (5)	
H2A	0.5293	0.7195	0.3373	0.043*	
C3	0.44280 (16)	0.4921 (3)	0.35755 (16)	0.0313 (5)	
C4	0.44120 (17)	0.2906 (4)	0.38632 (18)	0.0365 (5)	
H4A	0.3795	0.2324	0.3832	0.044*	
C5	0.53449 (17)	0.1771 (4)	0.42016 (18)	0.0355 (5)	
H5A	0.5359	0.0402	0.4397	0.043*	
C6	0.62590 (16)	0.2680 (3)	0.42492 (15)	0.0293 (4)	
S1	0.74353 (4)	0.11570 (9)	0.46843 (4)	0.03281 (19)	
O1	0.26690 (14)	0.5340 (3)	0.32361 (16)	0.0553 (5)	
O2	0.34762 (14)	0.7968 (3)	0.30119 (15)	0.0513 (5)	
C7	−0.08790 (19)	0.3289 (4)	0.33222 (18)	0.0377 (5)	
H7A	−0.1481	0.3087	0.3418	0.045*	
C8	0.07316 (18)	0.3574 (4)	0.35568 (19)	0.0386 (5)	
H8A	0.1473	0.3593	0.3885	0.046*	
N2	0.01283 (16)	0.3160 (3)	0.40391 (16)	0.0372 (4)	
H1NA	−0.141 (3)	0.397 (4)	0.185 (3)	0.057 (9)*	
N3	0.01514 (15)	0.3939 (3)	0.25866 (15)	0.0378 (5)	
N4	−0.08644 (15)	0.3754 (3)	0.24581 (16)	0.0356 (4)	
H1NB	0.033 (2)	0.284 (4)	0.467 (3)	0.056 (9)*	

### Atomic displacement parameters (Å^2^)

**Table:**

	*U*^11^	*U*^22^	*U*^33^	*U*^12^	*U*^13^	*U*^23^
O1W	0.0560 (12)	0.0645 (12)	0.0418 (10)	−0.0220 (9)	0.0277 (9)	−0.0133 (9)
O3	0.0481 (10)	0.0691 (12)	0.0361 (9)	0.0226 (9)	0.0128 (8)	−0.0110 (9)
O4	0.0309 (9)	0.0637 (12)	0.0627 (12)	−0.0006 (7)	0.0225 (8)	0.0041 (9)
O5	0.0381 (9)	0.0573 (10)	0.0332 (9)	0.0099 (7)	0.0131 (7)	0.0110 (8)
N1	0.0338 (10)	0.0511 (12)	0.0294 (10)	0.0095 (8)	0.0136 (8)	0.0016 (9)
C1	0.0303 (10)	0.0394 (11)	0.0397 (12)	−0.0027 (9)	0.0179 (9)	0.0001 (10)
C2	0.0378 (12)	0.0345 (11)	0.0376 (12)	0.0023 (9)	0.0188 (9)	0.0036 (9)
C3	0.0289 (10)	0.0382 (11)	0.0250 (10)	0.0054 (8)	0.0108 (8)	−0.0016 (9)
C4	0.0273 (10)	0.0432 (12)	0.0400 (13)	−0.0006 (9)	0.0163 (9)	0.0011 (10)
C5	0.0345 (11)	0.0352 (11)	0.0397 (12)	0.0022 (9)	0.0195 (10)	0.0049 (10)
C6	0.0284 (10)	0.0374 (11)	0.0229 (10)	0.0026 (8)	0.0126 (8)	−0.0001 (8)
S1	0.0276 (3)	0.0439 (3)	0.0256 (3)	0.0065 (2)	0.0110 (2)	0.0010 (2)
O1	0.0336 (9)	0.0730 (12)	0.0644 (13)	0.0111 (8)	0.0267 (8)	0.0157 (10)
O2	0.0509 (11)	0.0436 (10)	0.0545 (12)	0.0141 (8)	0.0199 (9)	0.0063 (9)
C7	0.0367 (12)	0.0416 (12)	0.0370 (12)	−0.0036 (10)	0.0188 (10)	−0.0047 (10)
C8	0.0335 (11)	0.0392 (12)	0.0407 (13)	−0.0020 (9)	0.0149 (10)	−0.0004 (10)
N2	0.0414 (11)	0.0373 (10)	0.0298 (11)	0.0010 (8)	0.0136 (8)	0.0021 (8)
N3	0.0375 (10)	0.0408 (10)	0.0369 (11)	−0.0075 (8)	0.0186 (8)	0.0011 (8)
N4	0.0312 (9)	0.0402 (10)	0.0309 (10)	−0.0045 (8)	0.0102 (8)	−0.0015 (8)

### Geometric parameters (Å, °)

**Table:**

O1W—H2W	0.950 (18)	C4—C5	1.386 (3)
O1W—H1W	0.917 (18)	C4—H4A	0.9300
O3—S1	1.4472 (18)	C5—C6	1.389 (3)
O4—S1	1.4411 (18)	C5—H5A	0.9300
O5—S1	1.4508 (18)	C6—S1	1.779 (2)
N1—O1	1.222 (3)	C7—N4	1.304 (3)
N1—O2	1.224 (3)	C7—N2	1.326 (3)
N1—C3	1.470 (3)	C7—H7A	0.9300
C1—C6	1.382 (3)	C8—N3	1.291 (3)
C1—C2	1.384 (3)	C8—N2	1.355 (3)
C1—H1A	0.9300	C8—H8A	0.9300
C2—C3	1.379 (3)	N2—H1NB	0.86 (3)
C2—H2A	0.9300	N3—N4	1.362 (3)
C3—C4	1.376 (3)	N4—H1NA	0.87 (3)
			
H2W—O1W—H1W	110 (3)	C5—C6—S1	118.29 (16)
O1—N1—O2	123.5 (2)	O4—S1—O3	113.45 (12)
O1—N1—C3	118.0 (2)	O4—S1—O5	112.82 (11)
O2—N1—C3	118.42 (19)	O3—S1—O5	112.42 (12)
C6—C1—C2	119.7 (2)	O4—S1—C6	106.58 (10)
C6—C1—H1A	120.1	O3—S1—C6	105.01 (10)
C2—C1—H1A	120.1	O5—S1—C6	105.75 (10)
C3—C2—C1	118.4 (2)	N4—C7—N2	107.0 (2)
C3—C2—H2A	120.8	N4—C7—H7A	126.5
C1—C2—H2A	120.8	N2—C7—H7A	126.5
C4—C3—C2	123.16 (19)	N3—C8—N2	111.8 (2)
C4—C3—N1	118.48 (19)	N3—C8—H8A	124.1
C2—C3—N1	118.35 (19)	N2—C8—H8A	124.1
C3—C4—C5	117.9 (2)	C7—N2—C8	106.2 (2)
C3—C4—H4A	121.0	C7—N2—H1NB	125 (2)
C5—C4—H4A	121.0	C8—N2—H1NB	129 (2)
C4—C5—C6	120.0 (2)	C8—N3—N4	103.57 (19)
C4—C5—H5A	120.0	C7—N4—N3	111.5 (2)
C6—C5—H5A	120.0	C7—N4—H1NA	128 (2)
C1—C6—C5	120.84 (19)	N3—N4—H1NA	121 (2)
C1—C6—S1	120.85 (16)		
			
C6—C1—C2—C3	1.4 (3)	C4—C5—C6—S1	−179.78 (17)
C1—C2—C3—C4	−2.0 (3)	C1—C6—S1—O4	16.1 (2)
C1—C2—C3—N1	177.05 (19)	C5—C6—S1—O4	−165.17 (17)
O1—N1—C3—C4	−0.7 (3)	C1—C6—S1—O3	−104.6 (2)
O2—N1—C3—C4	178.5 (2)	C5—C6—S1—O3	74.2 (2)
O1—N1—C3—C2	−179.8 (2)	C1—C6—S1—O5	136.35 (19)
O2—N1—C3—C2	−0.6 (3)	C5—C6—S1—O5	−44.87 (19)
C2—C3—C4—C5	1.1 (3)	N4—C7—N2—C8	0.1 (3)
N1—C3—C4—C5	−178.02 (19)	N3—C8—N2—C7	−0.3 (3)
C3—C4—C5—C6	0.5 (3)	N2—C8—N3—N4	0.3 (3)
C2—C1—C6—C5	0.0 (3)	N2—C7—N4—N3	0.0 (3)
C2—C1—C6—S1	178.76 (17)	C8—N3—N4—C7	−0.2 (2)
C4—C5—C6—C1	−1.0 (3)		

### Hydrogen-bond geometry (Å, °)

**Table:**

*D*—H···*A*	*D*—H	H···*A*	*D*···*A*	*D*—H···*A*
N4—H1NA···O5^i^	0.88 (4)	1.88 (4)	2.744 (3)	169 (2)
O1W—H1W···N3	0.91 (4)	2.17 (4)	3.041 (3)	160 (4)
N2—H1NB···O1W^ii^	0.86 (4)	1.84 (4)	2.692 (3)	171 (3)
O1W—H2W···O3^iii^	0.95 (4)	1.86 (4)	2.774 (3)	161 (5)
C7—H7A···O4^iv^	0.93	2.36	3.063 (3)	132.
C8—H8A···O1	0.93	2.54	3.186 (4)	126.

Symmetry codes: (i) *x*−1, −*y*+1/2, *z*−1/2; (ii) *x*, −*y*+1/2, *z*+1/2; (iii) −*x*+1, *y*+1/2, −*z*+1/2; (iv) *x*−1, *y*, *z*.
